# Breastfeeding Promotion, Support and Protection: Review of Six Country Programmes

**DOI:** 10.3390/nu4080990

**Published:** 2012-08-14

**Authors:** Nune Mangasaryan, Luann Martin, Ann Brownlee, Adebayo Ogunlade, Christiane Rudert, Xiaodong Cai

**Affiliations:** 1 United Nations Children’s Fund, 3 United Nations Plaza, New York, NY 10017, USA; Email: ogunladeb@yahoo.com (A.O.); crudert@unicef.org (C.R.); cai@unfpa.org (X.C.); 2 FHI 360, Washington, DC 20009, USA; Email: lmartin@fhi360.org; 3 School of Medicine, University of California, San Diego, CA 92093, USA; Email: absykora@gmail.com

**Keywords:** breastfeeding, infant feeding, IYCF, nutrition, equity

## Abstract

Reviews of programmes in Bangladesh, Benin, the Philippines, Sri Lanka, Uganda, and Uzbekistan sought to identify health policy and programmatic factors that influenced breastfeeding practices during a 10 to 15 year period. Exclusive breastfeeding rates and trends were analysed in six countries in general and from an equity perspective in two of them. Success factors and challenges were identified in countries with improved and stagnated rates respectively. The disaggregated data analysis showed that progress may be unequal in population subgroups, but if appropriately designed and implemented, a programme can become a “health equalizer” and eliminate discrepancies among different subgroups. Success requires commitment, supportive policies, and comprehensiveness of programmes for breastfeeding promotion, protection and support. Community-based promotion and support was identified as a particularly important component. Although health workers’ training on infant feeding support and counselling was prioritized, further improvement of interpersonal counselling and problem solving skills is needed. More attention is advised for pre-service education, including a stronger focus on clinical practice, to ensure knowledge and skills among all health workers. Large-scale communication activities played a significant role, but essential steps were often underemphasized, including identifying social norms and influencing factors, ensuring community participation, and testing of approaches and messages.

## 1. Introduction

Undernutrition, an important determinant of child health [1,2], is the underlying cause of 35% of the disease burden in children younger than 5 years [3]. At the same time, implementation of proven public health and nutrition interventions, including promotion of breastfeeding, has resulted in sizeable progress in improving child survival and health in many countries [4,5]. 

Optimal breastfeeding practices can help prevent undernutrition [6,7] during early childhood. Ensuring high rates of exclusive breastfeeding (“Exclusive breastfeeding” is defined as giving no other food or drink—not even water—except breast milk. The definition does allow the infant to receive oral rehydration salts (ORS), drops and syrups (vitamins, minerals and medicines) [8].) in the first 6 months of life and continued breastfeeding from 6 to 11 months is the preventative intervention with the highest potential among others to reduce under-5 deaths in the developing world [9]. In addition, optimal breastfeeding contributes to young child growth [4,10] and development [11,12,13]. 

However, recent data indicate that only about 36% of infants younger than 6 months are exclusively breastfed in developing countries [14]. Improving exclusive breastfeeding rates among the poorest may be particularly important in the reduction of global disparities in child survival and health [15,16]. 

Several global public health initiatives since the 1990s continue to positively influence countries around the globe to include optimal Infant and Young Child Feeding (IYCF) practices, especially the promotion, protection and support of breastfeeding (“Breastfeeding protection” means application of a policy of maternity entitlements and adoption of the International Code of Marketing of Breast-milk Substitutes and subsequent relevant health assembly resolutions; “promotion” means ensuring that all who are responsible for communicating with the general public provide accurate and complete information about breastfeeding; “support” means providing skilled counselling and support by the health system, as well as by the community [17].) as an integral part of their public health policies and programmes for improving child survival and health [18,19,20]. These global efforts stem from scientific and programmatic evidence which continues to show that ensuring optimal breastfeeding practices is one of the most cost-effective preventative strategies for improving child survival [9,21,22,23,24,25,26]. 

The United Nations Inter-agency Group for Child Mortality Estimation (IGME) estimates that under-five mortality at the global level declined from about 88 deaths per 1000 live births in 1990 to 57 in 2010 [27], with the rapid expansion of several basic public health and nutrition interventions including immunization, breastfeeding, vitamin A supplementation, and safe drinking water in successful countries [5]. However, emerging evidence shows that the highest under-five mortality rates are increasingly clustered in Sub-Saharan Africa and Southern Asia, especially in poor communities or households within these regions, and that children from rural and poorer households are more affected [5,27,28]. This underscores that a better understanding of disparities through disaggregated data analysis on breastfeeding practices is needed to improve them, with a focused approach to reach the most vulnerable groups. 

This paper presents the main findings from a review of country-level programmes to promote, protect, and support breastfeeding (referred to as “programmes” or “programme” in this article) in six countries over a period of 10 to 15 years (1993 to 2007). Each country review examined overall country programme progress at the national level which included numerous approaches and interventions with different scale and quality of implementation, documented important lessons learned, and provided recommendations for future programmes. The intent of this paper is not to make a comparative analysis of the six country programmes, but to summarize the findings by programme components and highlight overarching and commonly observed lessons and the programmatic implications that are applicable to these countries and beyond them. Detailed findings from each review, as well as the list of documents and detailed description of review activities in each country are available in separate country reports [29]. Further, using nationally representative data from Benin and Bangladesh (representing two different country contexts in terms of programme impact), we demonstrate the impact of these efforts to improve exclusive breastfeeding status in different socioeconomic subgroups. Our findings are discussed within the context of the successes, challenges and potential impact of breastfeeding in addressing the child survival and health disparities that currently exist among different population sub-groups.

## 2. Method

### 2.1. Country Selection and Context

In 2008, six countries—Bangladesh, Benin, the Philippines, Sri Lanka, Uganda, and Uzbekistan—were selected for study based on a number of criteria, such as (i) wide range of programmatic and policy experiences (both negative and positive); (ii) potential for significant cross-country learning from diverse demographic, health, nutrition, and development contexts (Table 1); (iii) geographic representation of different world regions; (iv) interest by international and national stakeholders involved in IYCF-related activities; and (v) availability of nationally representative breastfeeding data over the time period of interest. Even though country policies, strategies, and programmes often cover both breastfeeding and complementary feeding (IYCF) practices, the purpose of the review was to specifically focus on breastfeeding outcomes, primarily the exclusive breastfeeding rate of infants 0–5 months of age (<6 months) referred to in this article as “exclusive breastfeeding”. 

**Table 1:** Country context: selected demographic, health and development indicators for Bangladesh, Benin, Philippines, Sri Lanka, Uganda, and Uzbekistan.

Selected Indicators	Bangladesh		Benin		Philippines		Sri Lanka ^+^		Uganda		Uzbekistan ^++^	
**Demographic indicators ^#^**	**1995**	**2005**	**1995**	**2005**	**1995**	**2005**	**1995**	**2005**	**1995**	**2005**	**1995**	**2005**
Total population (millions)	118	141	5.65	7.63	69.3	85.6	18.2	19.8	20.8	28.4	22.9	26.0
Population children under 5 years (millions)	16.6	16.0	1.04	1.33	9.99	11.4	1.70	1.77	4.13	5.61	3.31	2.58
Urban population (%)	23.7	28.0	37.2	41.2	48.8	48.1	17.2	14.5	11.7	12.7	38.4	37.2
Child mortality indicators *												
Under 5 mortality rate	114	64.2	160	129	47.7	34.5	26.7	19.5	167	120	69.7	56.7
Infant mortality rate	80.8	49.1	97.5	80.5	34.8	26.6	22.5	16.7	99.7	74.7	57.5	47.8
Nutrition indicators **	**1996/1997**	**2007**	**1996**	**2006**	**1996**	**2003**	**1993**	**2006**	**1995**	**2006**	**1996**	**2006**
Stunting prevalence among children <5 years (%)	55	36	29	38	30	30	24	18	40	32	34	15
Underweight prevalence among children <5 years (%)	56	46	29	23	28	28	38	22	25	20	17	5
**Health indicators ¶**		**2007**		**2006**		**2008**		**2006/2007**		**2006**		**2006**
Antenatal care coverage, at least once (%)		51		84		91		99		94		99
Antenatal coverage, 4 times or more (%)		21		61		70		-		47		-
Health facility deliveries (%)		15		78		38		98		41		97
Deliveries attended by skilled health personnel (%)		18		74		62		99		42		100
Adult HIV prevalence (15–49 years)		<0.1		1.2		<0.1		<0.1		5.4		0.1
**Water and sanitation indicators ^¶¶^**		**2008**		**2008**		**2008**		**2008**		**2008**		**2008**
Percentage using improved drinking water		81		73		92		89		69		87
Percentage using improved sanitation facilities		53		12		72		91		34		100
**Human development indicators ^†^**												
Life expectancy at birth (years)		68		54.9		68		74.5		52.5		67.6
Adult literacy rate ages 15-older (%)		-		-		95.4		90.6		-		-
Human Development Index (HDI) value		0.484		0.419		0.635		0.676		0.430		0.624

Data sources: ^#^ Demographic indicators: United Nations, Department of Economic and Social Affairs, Population Division (2011), World Population Prospects: The 2010 Revision [30]; * Child mortality indicators: United Nations IGME Database [31]; ** Nutrition indicators: Demographic and Health Survey country reports [32]; ^+^ Sri Lanka data from the government database [33]; ^++^ Uzbekistan data from multiple indicators cluster survey [34]; ^¶^ Health indicators: United Nations Children’s Fund Database [35]; ^¶¶^ Water and sanitation indicators: World Health Organization/United Nations Children’s Fund Joint Monitoring Programme for Water and Sanitation [36]; ^†^ Human development indicators measure different aspects of well-being and human development, United Nations Development Programme [37].

### 2.2. The Review Process: Policy and Programming Experience

**Figure 1:**
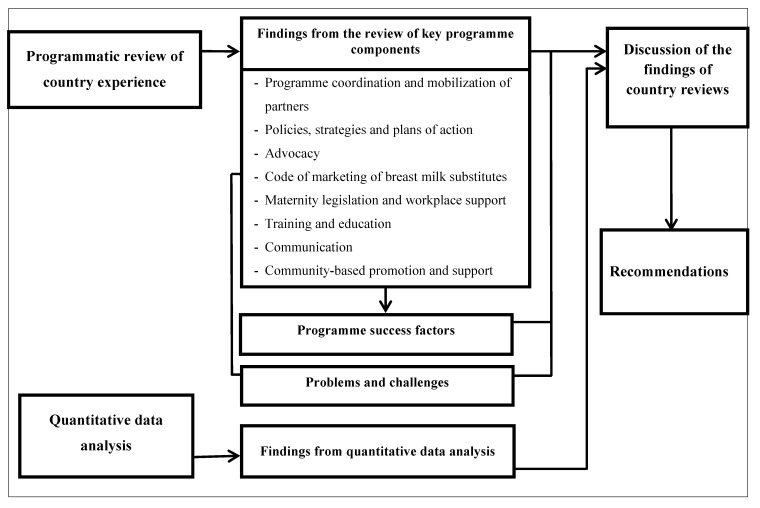
The conceptual framework guiding the review process.

The overall review process was guided by a conceptual framework (Figure 1). To ensure that a true reflection of the country-level policy and programming experience from 1993 to 2007 was portrayed and documented, a mix of approaches was employed including a literature review, analysis of relevant data, key informant interviews, site visits and stakeholder discussion workshops. Nearly 300 country reports, assessments, evaluations, and other documents were studied as part of the six-country review. 

A questionnaire was designed drawing on a previously developed tool for assessing national IYCF policies, programmes, and practices [38]. The qualitative semi-structured interview design was used to identify and explore a range of issues on programming across the six countries. In total, 137 interviews were conducted between July and November 2008 among key informants responsible for breastfeeding-related activities in the six countries. The key informants included representatives from government ministries (e.g., the Ministry of Health (MOH)) and health facilities, UNICEF and other United Nations (UN) agencies, civil society organizations (CSOs), as well as professional associations, development partners, health professionals, academicians and researchers. Visits to and observations of health facilities and project sites were undertaken. At the end of the country visits, workshops ranging from ~3 to 6 h with stakeholders involved with programme implementation were held to share findings from the review and elicit feedback. The programme review process was complemented with collection and review of relevant country reports, documents, and programme communication materials.

### 2.3. Quantitative Data Analysis

Nationally representative trend data on exclusive breastfeeding were acquired, with the first data points around 1993 to 1996 and the last data points around 2003 to 2007. The data were obtained from the Demographic and Health Survey (DHS) reports from the DHS website and UNICEF’s Childinfo global database A later exploratory exercise was conducted using DHS data from Benin and Bangladesh to examine the effects of country-level programme efforts on exclusive breastfeeding rates from an equity perspective. Disaggregating breastfeeding trend data by different population sub-groups could reveal population segments that might have benefited most or least from programme implementation, allowing more focused future planning towards accelerated and equitable progress. The selection of countries was based on availability of data for stratification and two different trends: stagnation (Bangladesh) and marked improvement (Benin).

## 3. Results

### 3.1. Findings from the Review of Selected Key Programme Components in 6 Countries

#### 3.1.1. Programme Coordination and Mobilization of Partners

The Global Strategy for IYCF [19] recognises the need for a national coordination structure and a wide range of partners to achieve substantial sustained impact [20,39]. In general, the national government through the MOH was the lead agency for programming in the reviewed countries, with a certain degree of involvement of other structures, except in Sri Lanka where the responsibilities for IYCF were mainly within the MOH units. In the Philippines, other government institutions involved included the Department of Interior and Local Government which mobilized local councils to prioritize IYCF in their plans. In Bangladesh the Ministry of Local Government, Rural Development and Cooperatives complemented the work of the Ministry of Health and Family Welfare and the Institute of Public Health Nutrition in all city corporations and municipalities [40,41]. In Benin the Ministry of Family and National Solidarity conducted nutrition-related work through its network of community outreach workers and social promotion centres, and in Uganda the National Bureau of Standards, Ministry of Trade, and Ministry of Justice had oversight responsibilities for national regulations on marketing of infant and young child foods. In Uzbekistan, the National Interdepartmental Committee on Nutrition (subordinate to the Cabinet of Ministers) was responsible for formulating action plans and fostering enabling conditions for disseminating policy on exclusive breastfeeding. 

Apart from the national government institutions, several partners from CSOs, research and training institutions, as well as development partners (UN organizations, international finance institutions, professional societies and bilateral agencies) were instrumental in providing a wide range of programming and policy support.

#### 3.1.2. Policies, Strategies and Plans of Action

A national IYCF policy is usually a formal document setting out the position of the government on recommended IYCF practices and the principles of action to achieve national goals for these practices [20]. Bangladesh, the Philippines and Sri Lanka were early adopters of supportive breastfeeding policies and the initiators of national breastfeeding promotion in the 1980s. Two global initiatives launched in the 1990s—the Innocenti Declaration on the protection, promotion and support of breastfeeding [18] and the Baby-Friendly Hospital Initiative (BFHI) [42]—gave impetus to policy work, as did WHO’s shift in 2002 from recommending 4 months of exclusive breastfeeding to 6 months as an international norm [19]. The speed and extent to which countries adopted the new 6 month recommendation differed considerably. For instance, effective advocacy by the Bangladesh Breastfeeding Foundation led to adoption of the 6 month recommendation by the MOH in 2003. In Sri Lanka, the recommendation caused confusion and met with resistance by health professionals initially, but with firm and sustained engagement by various individuals in the government, 6 months became official policy in 2006. 

National IYCF strategies and plans of action define the overarching public health goals and objectives of improving IYCF practices and identify key actions and actors to achieve the objectives [39]. Action plans in the 1990s often focused on BFHI alone with little attention to other key components such as health service initiatives beyond the hospitals, community-based counselling and support, and communication. Additionally, little consideration was given to scaling up on-going programmes, particularly successful community-based initiatives. However, the development and endorsement of the Global Strategy for IYCF [19] served as a strong motivation for countries to develop more comprehensive national strategies and begin to devise the approaches needed to increase coverage. The required time differed: in 2004 a task force in the Philippines was created to prepare a national strategy and by 2005 a national policy with a comprehensive plan of action was in place, while in Bangladesh, even though the process of developing a strategy started around the same time, a strategy was not adopted by the government until 2007.

#### 3.1.3. Advocacy

To gain political support for IYCF, breastfeeding advocates in the six countries used several approaches. The involvement of highly recognized national and international personalities added a strong voice to policy dialogue. In Bangladesh, Sri Lanka, the Philippines and Uganda, outstanding leadership was provided by dedicated paediatricians who helped advocate for legislation on maternity protection and a Code of Marketing of Breastmilk Substitutes. Furthermore, coalitions using a variety of strategies (e.g., celebrity involvement, intense media campaigns, and other events that created publicity) helped generate widespread support. An evidence-based advocacy strategy was applied in Benin and Uganda using the PROFILES tool (a process for nutrition policy analysis and advocacy that quantifies the consequences of malnutrition and the economic and human benefits of nutrition improvement and displays the results graphically) [43,44]. In addition, several countries used the Lancet child survival series [9,45,46] for evidence-based arguments.

#### 3.1.4. Implementation of the International Code of Marketing of Breastmilk Substitutes

Countries made efforts to give effect to the International Code of Marketing of Breastmilk Substitutes and subsequent Resolutions of the World Health Assembly which recommend adopting national laws or regulations, providing guidelines, and monitoring and enforcing compliance [47,48]. Sri Lanka, Bangladesh and the Philippines passed laws in 1981, 1984 and 1986 respectively. Benin and Uganda passed relevant laws in 1997. Uzbekistan does not have any laws or regulations on marketing of breast milk substitutes. In all cases, enforcement and effective monitoring proved challenging and difficult to implement and sustain, with very weak or no penalties in place for companies that persisted in violating regulations. 

National codes of marketing of substitutes diminished the influence of formula companies in the marketplace and health facilities, particularly in Bangladesh and Sri Lanka. The importance of on-going promotion of breastfeeding, laws to regulate the marketing of substitutes, and enforcement of these laws was particularly evident in the Philippines where the rate of exclusive breastfeeding dropped when breastfeeding promotion waned and the marketing campaigns of infant formula companies intensified. It took a multi-pronged intensive advocacy effort to strengthen the effectiveness of the Code there.

#### 3.1.5. Maternity Protection Legislation and Workplace Support

Provisions of the International Labour Organization (ILO) Maternity Protection Convention in 2000 [49] and the WHO recommendation of 6 months of exclusive breastfeeding [19] were used to advocate for revisions to existing maternity protection legislation and to raise awareness of the rights of women and their contribution to child health. All the countries reviewed mandated paid maternity leave in the public sector. The average duration of paid maternity leave ranged from 60 days in the Philippines to 2 years in Uzbekistan. The private and informal sectors were not adequately covered, posing challenges for breastfeeding women, while the 2000 ILO Maternity Protection states that it applies to “all employed women, including those in atypical forms of dependent work”, meaning not only women in public and private sectors, but also in jobs like domestic maids, cleaners or picking crops in the fields, *etc*. [50] These types of “informal” workers are often left out of maternity protection arrangements. Uzbekistan offered paid maternity leave for both private and public sector employees with full salary for 4 months and then twice the national minimum salary per month until the child reached 24 months. For public sector employees in Sri Lanka, the government provided full pay for the first 84 working days followed by an optional half pay for the next 84 days and then leave without pay up to 12 months of the child’s age. The paid maternity leave for private sector employees was limited to 12 weeks, covering only the first two children.

#### 3.1.6. The Baby Friendly Hospital Initiative (BFHI)

The BFHI was launched by WHO and UNICEF in 1991, following the joint WHO/UNICEF statement on breastfeeding in maternity services in 1989 [51] and the adoption of the Innocenti Declaration in 1990 [18]. The BFHI promotes implementation of the “Ten Steps to Successful Breastfeeding” to create an environment conducive for breastfeeding in health facilities [42]. The Initiative was launched in the 1990s in all six countries. In Bangladesh, Benin, the Philippines and Sri Lanka, work on the Initiative was initially intense and brought energy and enthusiasm. However, the failure to adequately monitor compliance, provide refresher training, and fully institutionalize the BFHI led to a decline in compliance with the “Ten Steps”. The emphasis on improving support for breastfeeding in health facilities was most appropriate in Benin, Sri Lanka, and Uzbekistan where from three-fourths to almost all women deliver in health institutions. Overall, the BFHI faced challenges related to sustainability and integration, national ownership, monitoring and reassessment and the link with the community. 

#### 3.1.7. Training and Education

Building and strengthening the capacity of health care providers was a major component of programmes in all six countries. This took three forms:

1. *In-service training* updated knowledge and skills and motivated staff. The WHO 40-h breastfeeding counselling course and the WHO/UNICEF 18-h BFHI course were the primary curricula used for in-service training between 1995 and 2005, with tens of thousands of health care providers trained through these courses.
2. *Pre-service education* helped ensure that upcoming/future health care providers were equipped with correct information and practical skills to provide IYCF counselling and support. In most of the countries reviewed, pre-service education focused primarily on theoretical knowledge, without much practical guidance for dealing with lactation management issues. Moreover, in many cases messages were not always harmonized in different curricula (e.g., the curricula for nurses and doctors).
3. *Professional development courses* provided in-depth training to individuals with major responsibilities for IYCF in health facilities, academic institutions and government agencies. A one-month course on Breastfeeding Practice and Policy at the Institute of Child Health in London and a one-month lactation management course at Wellstart International in San Diego trained a generation of professionals who remained breastfeeding advocates for decades. For instance, participants from Uganda started a breastfeeding CSO and set up the first lactation clinic in sub-Saharan Africa. Professional development courses in lactation management were instrumental in training health professionals who became focal points and master trainers in lactation management in the Philippines, Sri Lanka, and Uzbekistan.

#### 3.1.8. Communication

Communication on breastfeeding is essential for large-scale behavioural and social change and should be an intrinsic component of any national child survival, nutrition and health intervention [39]. Improving breastfeeding practices requires the use of multiple channels to reach priority audiences with context-specific messages. Interpersonal communication during home visits, group discussions and counselling sessions at health or community centres allow health providers and community workers to communicate specific messages, address individual concerns, problem-solve and support good practices. 

Formative research methods such as trials of improved practices [52] were used in Benin and Uganda to tailor messages to the specific contexts of the target audiences. In Bangladesh, the Philippines, Sri Lanka and Uzbekistan, the use of print media (leaflets, posters, newspaper, pamphlets and other printed materials) was a feasible communication channel, mainly because of high literacy rates and few main languages. However, printed materials were not as effective in Benin and Uganda because of low literacy rates and multiple languages. 

Other communication channels used in all countries, although with different degrees of coverage, included mass media (television, radio, and films), events such as World Breastfeeding Week, seminars or workshops for religious leaders and interpersonal communication. At the time of this review, messages through television were likely to reach more women in countries such as the Philippines (with 82% of women reporting that they watched television at least once a week) than in Uganda (with only 11% watching television at least once a week) [53,54]. 

#### 3.1.9. Community-Based Promotion and Support

Community-based promotion and support was the least developed component of most programmes. Community resources tapped for promotion and support included public health midwives in Sri Lanka, community nutrition workers in Benin and Bangladesh, community growth promoters in Uganda, peer counsellors in the Philippines and Benin and women leaders in the community in Uzbekistan. At the time of the review, the vast majority of the population in Bangladesh, the Philippines and Uganda remained untouched by community-based breastfeeding activities. 

Breastfeeding rates reflect the degree to which programmes took advantage of various opportunities for promoting and supporting breastfeeding by health and community workers and achieving scale. In Sri Lanka public health midwives had multiple contacts with pregnant and postpartum women in their homes and in the clinic as part of a continuum of care. In Uganda, however, most women did not have any postpartum contacts with health workers, which greatly limited the impact on feeding practices. Although multiple contact points existed, some programmes tended to focus almost entirely on growth monitoring while overlooking other opportunities to counsel and support mothers. 

### 3.2. Key Challenges

Key challenges to substantial progress in improving breastfeeding practices are summarized in Table 2. In countries with little or no progress in exclusive breastfeeding rates, barriers included poor infant feeding knowledge and skills coupled with detrimental cultural beliefs and practices. Lack of adequate counselling and support to caregivers by trained personnel at the health facility and community level was an important factor limiting reach and effectiveness. In Bangladesh, Benin, Uganda and the Philippines, high staff turnover, poor supervision, missed opportunities for counselling and support, and other weaknesses in the health delivery system limited gains that could have been achieved. Weak political commitment leading to poor allocation of human, financial and governmental/administrative resources for effective promotion, protection and support of breastfeeding was a major issue identified at the national and sub-national levels.

**Table 2:** Challenges and obstacles to adoption of recommended breastfeeding practices.

Levels	Main Challenges and Problems Identified	
**Household/Individual**	**Lack of infant feeding knowledge and skills among caregivers**	
	-	Lack of knowledge of benefits of breastfeeding and the importance of exclusive breastfeeding.
	-	Assumption that breast milk is not enough to nourish infants.
	-	Lack of infant feeding skills, such as proper positioning and attachment and appropriate complementary feeding.
	-	Lack of understanding that insufficient milk is due to poor suckling techniques and not feeding frequently enough.
	**Lack of family support**	
	-	Extended family members encouraging mothers to give other liquids and foods early.
	-	Family members not able to support mothers through help with household tasks or other children.
	**Cultural beliefs and practices**	
**Health facility/Community**	-	Prelacteal feeds, delayed initiation, and discarding of colostrum.
	-	Giving water, herbal teas, watery porridges, and other drinks within the first six months.
	-	Using feeding bottles and various breastmilk substitutes.
	-	Poor complementary feeding practices such as delaying introduction beyond six months of age and/or giving foods with insufficient variety, energy density, or feeding frequency.
	**Unsupportive health facility and community-based services**	
	-	Health facility practices not conducive to the establishment of good breastfeeding practices.
	-	Limited knowledge on IYCF and lactation management, complementary feeding, and counseling skills among health providers and community volunteers.
	-	Lack of time to provide the needed IYCF support by the health providers and community volunteers.
	-	Poor supervision and monitoring of staff and volunteers trained to provide IYCF support.
**National/Sub-National**	**Unsupportive work environment**	
	-	Limited or no maternity leave.
	-	Inflexible working hours and lack of breastfeeding breaks.
	-	No breastfeeding rooms or space for expressing and storing breast milk.
	**Commercial pressures**	
	-	Widespread advertising of breastmilk substitutes through print media, radio, television, and billboard advertisements.
	-	Provision of gifts and incentives to influence health workers to promote formula products.
	-	Lack of monitoring and enforcement of marketing regulations for breastmilk substitutes.
	**Administrative and political challenges**	
	-	Weak national commitment to IYCF and nutrition and inadequate resources.
	-	Poor coordination among government offices and partners.
	-	Lack of integrated, cost-effective and sustained approaches to address health and nutrition needs.
	-	Rapid turnover of administrative, health service, as well as, community staff and volunteers with IYCF skills.
	-	Small-scale and fragmented community-based services.

### 3.3. Success Factors

Within the context of this study, success was defined in terms of improving and sustaining high exclusive breastfeeding rates of infants at 0–5 (<6) months of age. The multi-country review identified and summarized factors that positively influenced the exclusive breastfeeding rate over the 10 to 15-year period (Table 3). At the community level, breastfeeding promotion and support was most successful when there was community ownership, in which community leaders and respected members of the society were involved at all stages of the programme. In Benin, the involvement of community leaders and mothers in formative research and planning led to active efforts in mobilizing other community members to participate in implementing identified activities. 

Improving the skills of health workers in countries with high rates of institutional delivery was crucial for success. Sri Lanka’s well-established health structure contributed to its achievements. Public health midwives served as the main link between the health facility and the community, providing antenatal care including breastfeeding counselling and support in homes and clinics.

An enabling environment for breastfeeding at the national level through supportive national policies, effective programme development and implementation, and continued advocacy were also instrumental in improving and sustaining programme outcomes. 

**Table 3:** Factors for successful programming.

Levels	Main Programme Success Factors Identified
**Community **	- Community outreach and engagement of community leaders. - Interpersonal counselling and problem-solving skills of health providers and community workers to foster trial and adoption of improved feeding practices. - Formative research to develop a continuous, comprehensive communication strategy on IYCF that uses multiple channels and addresses specific barriers to optimal practices.
**Health facility **	- Timed and targeted IYCF counselling by health workers at critical times when mothers make feeding decisions and require support. - Effective implementation of the “Ten Steps for Successful Breastfeeding” in countries with high levels of institutional deliveries. - Pre-service education on key aspects of IYCF, including adequate clinical practice, to strengthen health workers’ IYCF-related knowledge and skills.
**Sub-national/National**	- Engagement of a diverse set of partners. - Integration of IYCF into existing programme platforms. - Continuous, effective leadership. - An appropriate balance and coordination of policy/advocacy, health services, and community-based interventions aimed at achieving results, scale, and sustainability. - Respected, dedicated and trustworthy champions of breastfeeding. - Evidence-based advocacy to address lack of awareness, complacency, controversy, and competing priorities. - National codes of marketing of breast milk substitutes with strong monitoring and enforcement mechanisms to diminish the influence of infant formula companies.
**International**	- International leadership through policy and programmatic guidance (e.g., Innocenti Declaration on the Protection, Promotion and Support of Breastfeeding, the Baby Friendly Hospital Initiative, and the Global Strategy for IYCF).

### 3.4. Effect of Programme on Trends in Exclusive Breastfeeding

**Figure 2:**
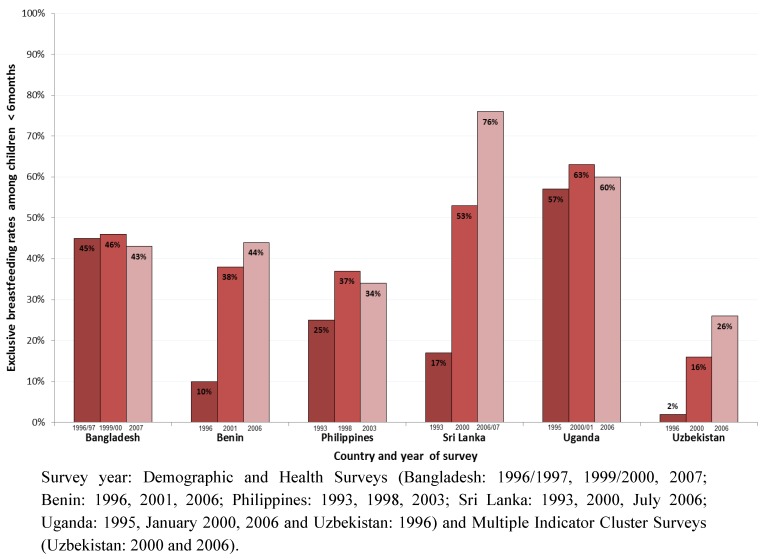
Exclusive breastfeeding rates: Trends in the six countries based on three recent surveys.

Figure 2 shows trends in exclusive breastfeeding rates among infants <6 months in the six countries over a period of 10 to 15 years. At the first data point (around 1993 to 1996), Benin (10%), Sri Lanka (17%) and Uzbekistan (2%) had the lowest exclusive breastfeeding rates among the six countries, and Bangladesh (45%) and Uganda (57%) had the highest rates. At the third data point (around 2003 to 2007), exclusive breastfeeding rates in Benin, Sri Lanka and Uzbekistan had increased to 44%, 76% and 26%, respectively with gains ranging from 24 percentage points in Uzbekistan and 34 percentage points in Benin to 59 percentage points in Sri Lanka (Figure 2). The countries that started with the highest exclusive breastfeeding rates—Bangladesh (43%) and Uganda (60%)—demonstrated the least improvement.

The lack of sizeable improvement in exclusive breastfeeding rates in Bangladesh, the Philippines and Uganda, with low institutional delivery rates of 15%, 38% and 41%, respectively (Table 1) can be explained in part by the limited attention to community promotion and support for breastfeeding coupled with administrative and political challenges as indicated in Table 3. In Benin, with institutional delivery rates of 78%, the BFHI, along with support for community-based counselling and large scale communication likely contributed to improvement in the exclusive breastfeeding rate; however, further progress may have been hindered due to lack of emphasis on breastfeeding at the national policy level combined with lack of adequately trained health professionals to provide skilled support (Table 2). Sri Lanka, with its well-developed health system, high rates of antenatal care and delivery in health facilities (95%), extensive lactation management training, and effective home visiting and outreach programmes, recorded the most significant gains in the exclusive breastfeeding rate. 

It should be however noted that since this review, which assessed the programmes during the time period of 1993–2007, extensive efforts to scale up breastfeeding promotion, protection, and support were undertaken in reviewed countries. The latest surveys, such as recent Bangladesh DHS survey, may provide new data reflecting the results of these efforts [55].

### 3.5. Effect of Programme on Exclusive Breastfeeding Trends by Selected Population Subgroups in Benin and Bangladesh

From policy and programme implementation standpoints, it matters whether the gains or improvements in breastfeeding outcomes are equitably distributed. A recent study revealed that the national averages of child survival and other child health indicators may conceal broad and widening health inequalities within many countries [28]. 

**Figure 3:**
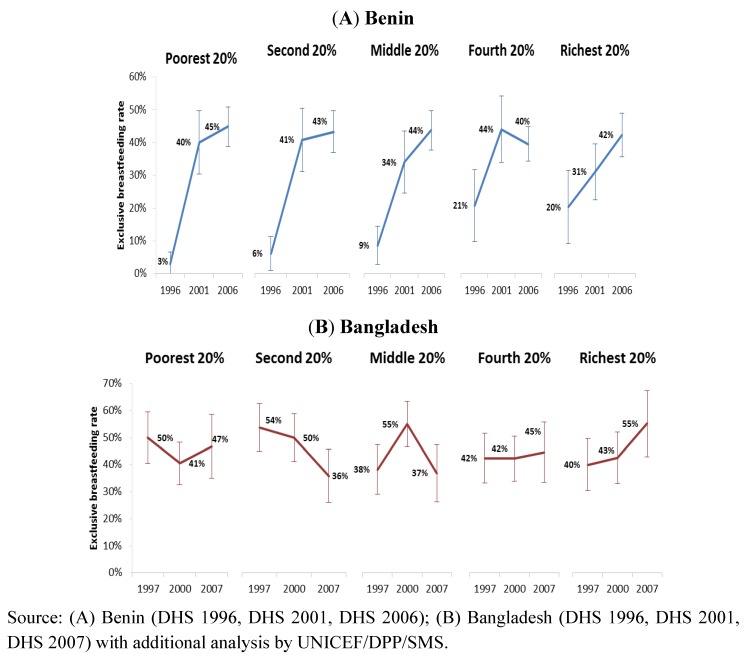
Trend of exclusive breastfeeding rate among infants <6 months by household wealth level, in Benin (**A**) and Bangladesh (**B**).

**Figure 4:**
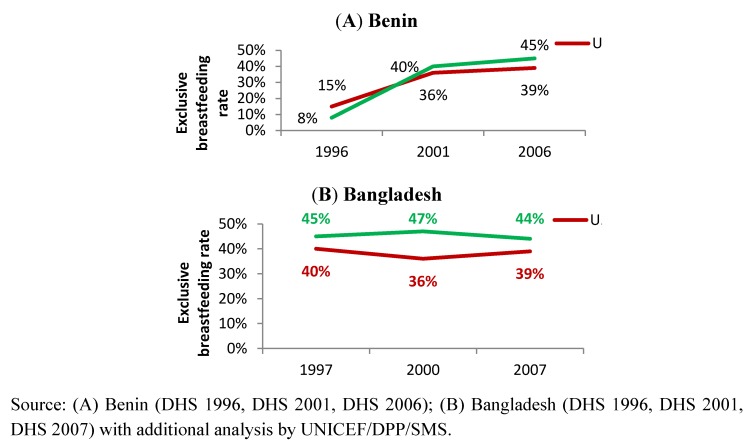
Trend of exclusive breastfeeding rate among infants <6 months, by residence in Benin (**A**) and Bangladesh (**B**).

**Figure 5:**
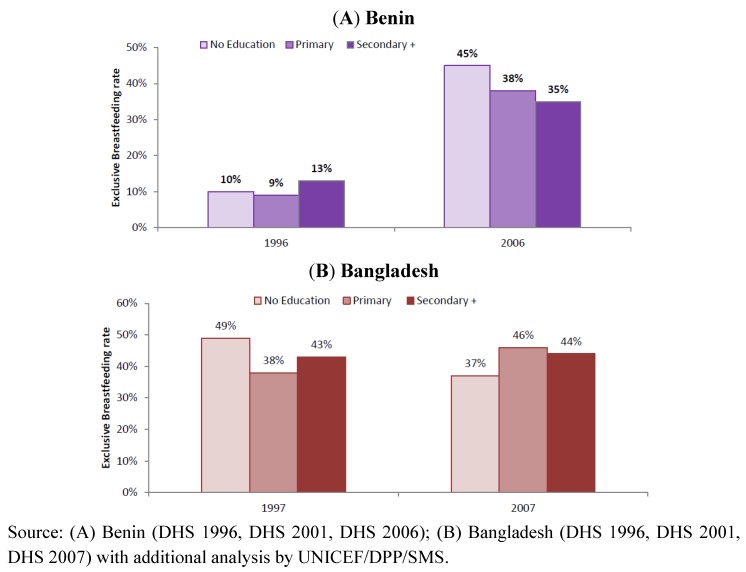
Trend of exclusive breastfeeding rate among infants <6 months, by maternal education level in Benin (**A**) and Bangladesh (**B**).

**Figure 6:**
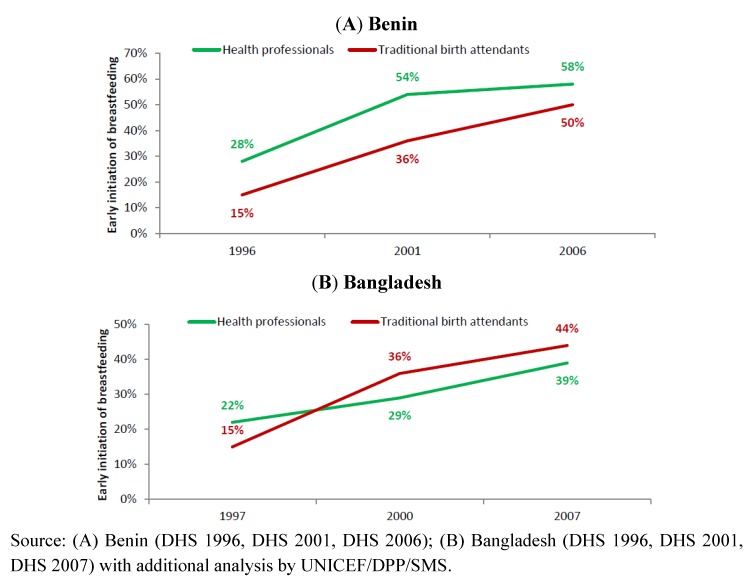
Trend of early initiation of breastfeeding (proportion of infants put to the breast within one hour of birth), by type of delivery assistance (health professionals versus traditional birth assistants) in Benin (**A**) and Bangladesh (**B**).

To demonstrate the importance of stratified analysis for assessment of progress and informing future programme development, we reviewed trends in exclusive breastfeeding rates among infants <6 months in Benin and Bangladesh by stratifying according to household wealth level (Figure 3), residence (Figure 4) and mother’s education level (Figure 5). The objective was to use examples of two countries with different trends to demonstrate the value of such analysis to identify priorities for future programming. Figure 6 illustrates the trends of early initiation of breastfeeding (proportion of infants put to breast within one hour of birth) by type of delivery assistance: health professional versus traditional birth attendant. 

The improvement in exclusive breastfeeding rates in Benin from 1996 to 2006 benefited all wealth levels and led to the elimination of large differences between rich and poor, with rates increasing from 3% to 45% among infants from the poorest households and from 20% to 42% among the richest (Figure 3). Thus, the Benin programme, through improved access to counselling and support services at the community level, supported the role of breastfeeding as a universal equalizer [56]. In Bangladesh, while the overall trends show a stagnant situation, the changes in exclusive breastfeeding from 1997 to 2007 favoured the richest 20% compared to the other households. Low institutional delivery rates (15%) and limited community promotion and support along with other challenges at the time of the review may explain the lack of improvement in those population groups in Bangladesh that did not have access to health care facilities. 

Both urban and rural women in Benin experienced marked improvements in exclusive breastfeeding rates with an estimated increment of about 37 percentage points among rural and 24 percentage points among urban women (Figure 4A). Although the overall improvement can be attributed to several of the key programme components listed in Figure 1, the higher rates among rural women may be an outcome of strong community-based promotion and support for breastfeeding in the rural communities. For instance, women’s support groups and community volunteers played an active role in breastfeeding promotion and acted as the interface between the community and the health system [57,58]. 

Little change in a decade in exclusive breastfeeding rates among mothers who lived in rural and urban areas of Bangladesh (Figure 4B) suggests that comprehensive and at-scale targeting both population groups may be required. Programming with strong community-based components is required to ensure that the rural population, with negative trends, is covered by effective interventions.

Maternal educational level has been identified as an important determinant of breastfeeding in many countries [59,60,61]. Our review revealed that exclusive breastfeeding rates improved dramatically among all mothers in Benin irrespective of their educational status; the largest improvements were observed among women with little or no education (Figure 5A). The improvement across all maternal education levels in Benin may be attributed to strong, large-scale communication activities on breastfeeding that were part of the health and nutrition strategies in the country during the period reviewed [62]. 

In Bangladesh, some improvement in practices by women with primary education (increase of 8 percentage points) is combined with reduced rates by women with no education (reduction of 12 percentage points), as shown in Figure 5B. This might be explained in part by less access to information and less attention to community-based interventions for counselling and support for those deprived of access to education.

Early initiation of breastfeeding rates improved dramatically in both countries whether deliveries were assisted by health providers or traditional birth attendants (Figure 6). However, there is significant room for further improvement, especially taking into consideration the impact of early initiation of breastfeeding on infant survival [24,63]. 

## 4. Discussion and Recommendations

Findings and lessons emerging from the review of six country programmes allowed us to derive to numerous conclusions and formed the basis for recommendations [62,64,65,66,67,68]. The review suggested that in various contexts breastfeeding practices can change through supportive policies and comprehensive programmes, coordinated efforts, skilled service providers and community workers, community outreach and an effective, multi-channel communication strategy. Few examples, however, exist of national-scale programmes. In many cases sustainability of initially successful programmes was hindered by failure of planners to integrate promising interventions into the health system and institutionalize them. New initiatives and an infusion of resources for other public health interventions at times pushed infant feeding off the agenda, leaving policies partially enacted and programmes under-resourced. 

The tendency has been to focus only on one or two components of a programme. Piecemeal approaches and ad hoc activities leave major barriers to improved practices unaddressed and fail to reach critical populations. Responsibility for infant feeding programmes is often spread among different stakeholders with lack of clarity on roles and responsibilities and inefficient use of resources. Lack of concerted and unified action among government and development partners results in lost opportunities to extend reach and coverage, economize, harmonize messages and learn from each other.

Protection, support and promotion—all are important to improving breastfeeding. The frequent use of the term “breastfeeding promotion” without “support” and “protection” may be interpreted by some to mean only communication and information sharing and not convey the important role of support and protection in improving breastfeeding practices.

The operational targets set in the Innocenti Declaration on the Protection, Promotion and Support of Breastfeeding aimed to create an enabling environment for breastfeeding in the workplace, marketplace and health facility. Some of the individuals interviewed as part of this review had dedicated decades to the enactment of these operational targets. Their advocacy, passion, and persuasive skills helped to move the agenda globally and nationally. 

What effect did the targets set by the Innocenti Declaration have on exclusive breastfeeding rates in the six countries reviewed? Maternity protection legislation likely had a relatively small effect on breastfeeding rates since most of the legislation applied to a very small percentage of women. National Codes on marketing of breastmilk substitutes were prioritized in most of reviewed countries, but their enforcement and monitoring is still a challenge that does not provide appropriate protection of breastfeeding.

The Baby-Friendly Hospital Initiative was actively implemented in many countries in the early years due to intensive advocacy by UNICEF and WHO and the dedicated work of breastfeeding advocates. However, insufficient effort to integrate the BFHI into the norms and requirements of national health systems and to institute mechanisms for on-going refresher training, monitoring and reassessment led to slippage in compliance and decreased focus on the BFHI. In addition, in countries such as Bangladesh and the Philippines with their high rates of home deliveries, inadequate attention was given to supporting women who delivered outside of health facilities. With the main focus on maternity services, knowledge and skills at the primary health care system were insufficiently addressed. Failure to provide skilled support in primary health care services and at community level will limit progress in improving breastfeeding practices. 

Even in countries where institutional delivery coverage is high, continued counselling and support is needed after discharge. This support is best delivered during multiple maternal and child health contacts in the primary health care system to maximize opportunities for support by capable staff. The task of infant feeding counselling and support should be integrated into the standard tasks and job descriptions of relevant health system staff, into the performance monitoring systems, as well as included in reporting such as that in child and maternal health cards, health centre registers, and district summary reports. Support and counselling by primary health care services should be complemented by support and counselling by community workers in areas with few or no health providers. 

The review showed that even though health workers’ training on infant feeding support and counselling was prioritised in countries at different levels and scale, further improvement of interpersonal counselling and problem solving skills was needed. Moreover, the training activities were not necessarily embedded in a comprehensive programme that included on-going mentoring, supportive supervision, and management information systems to ensure quality services. More attention was also needed for pre-service education, including clinical practices, to ensure high quality knowledge and skills among all health workers. 

Large-scale communication activities served to raise awareness of the importance of breastfeeding at all levels of society. However, there was room for improvement in the design and implementation of the communication strategies. Various essential processes were rarely emphasized, including identifying social norms and major influencing factors, ensuring active community participation and testing of relevant approaches and messages. 

National commitment can be significantly influenced by global initiatives to mobilize support for improved nutrition and infant feeding. Reviewed countries prioritized exclusive breastfeeding up to 6 months as an important indicator of success to improve child survival and nutrition due to global attention to this practice. In addition, continued breastfeeding should also be emphasized, as the evidence on the importance of breastfeeding beyond six months is compelling [3,16,63,69,70,71,72]. Breastmilk not only helps prevent disease and improve health outcomes, it is a source of nutrients, in others words, a “natural safety net” that extends beyond the first 6 months. Breastfeeding also brings long-term benefits such as lowered risk of higher blood pressure and blood cholesterol, obesity, asthma and certain cancers in adulthood [12,73] which should be emphasized as countries face increasing levels of non-communicable diseases. 

Additional analysis from Benin and Bangladesh showed that national programmes to improve breastfeeding do not necessarily address all population groups equitably. Benin was more successful in designing and implementing a multi-pronged programme that succeeded in lessening health inequities in the area of exclusive breastfeeding. Disaggregated analysis by different determinants (education level, economic status, geographic area, delivery assistance, *etc.*) is needed to identify the population groups that are most in need and tailor strategies to address their problems based on formative research. Equity analysis needs also to take into account other enabling and impeding factors in different population groups such as exposure to marketing of breastmilk substitutes. If appropriately designed and implemented, a successful programme will become an “equalizer’ and eliminate discrepancies between different population subgroups. Our study again confirmed that breastfeeding is a “safety net” against poverty if quality programmes are designed and implemented.

James P. Grant, former Executive Director, UNICEF, in State of the World’s Children 1985 says:

“Exclusive breastfeeding goes a long way towards cancelling out the health difference between being born into poverty or being born into afﬂuence. It is almost as if breastfeeding takes the infant out of poverty for those few vital months in order to give the child a fairer start in life and compensate for the injustices of the world into which it was born.”

## 5. Conclusions

The six-country review confirmed the need for a comprehensive strategy in accordance with the Global Strategy for IYCF [19]. The following eight key recommendations emerged from the review:

1. Develop and implement a comprehensive strategy that aims from the outset to achieve scale with specific tailored approaches to reach higher risk groups.
2. Establish and support a functional and sustainable coordination mechanism.
3. Foster an enabling environment by developing an evidence-based advocacy strategy; strengthening Code legislation and enforcement; extending maternity protection, and improving conditions in the workplace for breastfeeding women.
4. Strengthen the organizational and technical capacity for IYCF to ensure sustained ownership of IYCF programming and integrate IYCF within pre-service curricula and continuing and in-service education programmes.
5. Make the Ten Steps of the BFHI an integral component of the norms and standard operating procedures of facilities and incorporate them in accreditation procedures.
6. Extend IYCF counselling and support beyond maternity services and ensure that IYCF is addressed at appropriate contact points during pregnancy and throughout the first two years of life both within the health system and the community.
7. Scale up community-based interventions by building coalitions to achieve scale, using multiple platforms at the community level, equipping community workers with IYCF counselling and problem-solving skills and providing supportive supervision and mentoring.
8. Implement comprehensive communication strategies based on formative research on enablers and barriers to improved practices including lack of information and support, engrained detrimental cultural practices and social norms, workplace restrictions, and limited decision-making authority by women.

## Implications

The experiences of the six countries examined in this review can help inform current efforts to protect, promote, and support breastfeeding and raise well-deserved global attention to child nutrition. It is important that all global and country nutrition initiatives emphasize not only breastfeeding promotion, but also support and protection. Both exclusive breastfeeding up to 6 months and continued breastfeeding after 6 months lead to important health benefits and should be emphasized to measure national progress. Disaggregated data analysis can assist in fine-tuning a focused approach to reach population groups with the highest rates of sub-optimal practices, so that programmes can become a “health equalizer”, eliminating discrepancies among different population subgroups.
